# The Charming World of the Extracellular Matrix: A Dynamic and Protective Network of the Intestinal Wall

**DOI:** 10.3389/fmed.2021.610189

**Published:** 2021-04-16

**Authors:** Simona Pompili, Giovanni Latella, Eugenio Gaudio, Roberta Sferra, Antonella Vetuschi

**Affiliations:** ^1^Department of Biotechnological and Applied Clinical Sciences, University of L'Aquila, L'Aquila, Italy; ^2^Department of Life, Health and Environmental Sciences, Gastroenterology Unit, University of L'Aquila, L'Aquila, Italy; ^3^Department of Anatomical, Histological, Forensic Medicine, and Orthopedic Sciences, Sapienza University of Rome, Rome, Italy

**Keywords:** bowel, intestinal wall, extracellular matrix, basement membrane, interstitial matrix, MMPs, TIMPs

## Abstract

The intestinal extracellular matrix (ECM) represents a complex network of proteins that not only forms a support structure for resident cells but also interacts closely with them by modulating their phenotypes and functions. More than 300 molecules have been identified, each of them with unique biochemical properties and exclusive biological functions. ECM components not only provide a scaffold for the tissue but also afford tensile strength and limit overstretch of the organ. The ECM holds water, ensures suitable hydration of the tissue, and participates in a selective barrier to the external environment. ECM-to-cells interaction is crucial for morphogenesis and cell differentiation, proliferation, and apoptosis. The ECM is a dynamic and multifunctional structure. The ECM is constantly renewed and remodeled by coordinated action among ECM-producing cells, degrading enzymes, and their specific inhibitors. During this process, several growth factors are released in the ECM, and they, in turn, modulate the deposition of new ECM. In this review, we describe the main components and functions of intestinal ECM and we discuss their role in maintaining the structure and function of the intestinal barrier. Achieving complete knowledge of the ECM world is an important goal to understand the mechanisms leading to the onset and the progression of several intestinal diseases related to alterations in ECM remodeling.

## Introduction

The morphogenesis and homeostasis of the gastrointestinal tract are intimately linked to interactions between epithelial cells, arising from embryonic endoderm, and the stromal cells derived from mesenchyme. These cells contribute to the production and organization of the extracellular matrix (ECM). The extracellular matrix forms a complex network of proteins that not only acts as a support structure for resident cells but also interacts with them by modulating their phenotypes and functions. The resident cell, in turn, secretes numerous molecules that cooperate with the various components of the ECM, creating a specific local microenvironment.

The luminal surface of the intestinal mucosa (luminal side) is delimited by a monolayer of epithelial cells (enterocytes, enteroendocrine cells, goblet cells, and Paneth cells), which is an essential barrier against the external environment (1, 2). In this context, the ECM provides protection and mechanical support to cells conferring the elasticity and resilience to tensile forces to the organ.

The heterogeneous spatial and biochemical composition of the ECM modulates and synchronizes many cell functions and releases molecules such as integrins, cytokines, chemokines, and growth factors (3–5). The extracellular matrix and epithelial cells exchange biological and physical information to orchestrate organ function, showing that the ECM is not a static scaffolding, but rather a dynamic structure. Special ECM rearrangements build a specialized compartment in which unique cellular processes occur, such as the crypts and cradles of the intestinal stem cell pool. This niche nourishes the intestinal stem cells, which is pivotal for the self-renewal of the epithelium and tissue regeneration from injury (2, 6).

In this review, we give an overview of the intestinal ECM, describing its main components and their physiological functions.

## The Long History of the Extracellular Matrix

In the scientific community, the ECM is considered the most complex structural organization of tissues in organisms. To date, the paradigm “no cells, no ECM” persists, but it was a long way to go to prove it, as the cells were discovered even thousands of years later than the ECM (7). Around the 1700's, we believed that tissues and organs were composed of different forms and arrangements of connective tissue fibers and arose spontaneously. For many years this “fibers theory” represented the most accepted explanation of the basis of life, and it took ~100 years for this view to change. In the early 1800's, Lorenz Oken formulated the hypothesis “*omne vivum ex vivo*,” which means that “life may originate exclusively from something already alive” (7). An important discovery milestone in this field was made by Rudolf Virchow that, in the 1850's, shocked the scientific community with his hypothesis “*omnis cellula ex cellula*,” asserting that there are no cells without other cells (7). The revolutionary assertion that “cells are life and make fibers” took about 50 years until being generally accepted, and only subsequently was attention shifted to the discovery of the relationship between cells and intercellular space composition.

At the end of the 19th century, through light microscopy and then by innovative chemical and physical methods, the identification of collagen and elastin fibers, as well as the observation of several macromolecules in the intercellular compartment, was possible, and the unique definition to denote the subcellular space became the “extracellular matrix.” In the following years, the discovery of even more effective analysis and instruments allowed us to constantly highlight new details of ECM components (Table 1) (7). The period of 1930–1973 yielded several important discoveries for the connective tissue characterization and mainly for collagen and elastin—two core components of ECM. Particularly, electron microscopy and X-ray diffraction, leading to other increasingly advanced techniques, made it possible to identify and quantify amino acid sequences of proteins forming collagen and elastin fibers. Finally, in the last 40 years, a breakthrough revealed the active role of the ECM in cellular regulation, and new research in these fields demonstrated that the ECM directly influences the functions of the resident cells. A fine-tuned crosstalk between epithelial cells, mesenchymal cells, and ECM components is an essential step for the regulation of several key processes, such as cell adhesion, proliferation, differentiation, and apoptosis. Furthermore, the ECM exerts not only structural support for the cells and a physical barrier against the external microenvironment but represents a reservoir of growth factors involved in the activation of molecular pathways that regulate cell behavior. In this context, the biological functions of ECM are constant (1–10).

**Table 1:** Timeline of our understanding of the extracellular matrix (ECM).

**Date**	**Information**	**References**
1700	“Fiber theory”: tissues and organs are formed by connective tissue arose spontaneously	(7)
1809	Life can originate only from another life	(7)
1850	“Cellular theory”: cells do not exist without other cells	(7)
~1900	Collagen and elastin fibers identification	(7)
1930–1973	Connective tissue characterization	(7)
Last 40 years	Biological functions of the specific ECM components	(1–10)

## The Two Compartments of ECM: Basement Membrane and Interstitial Membrane

The ECM is composed of a complex and fine organized network of proteins and polysaccharide molecules, known as glycosaminoglycans (GAGs), and GAGs linked to protein forming proteoglycans (PGs). With ~300 different molecules interacting in the building of this amazing organization, the ECM represents an essential support structure for cells, tissues, and organs (4, 11). GAGs and PGs interact with several growth factors and ECM proteins and are involved in the regulation of cell proliferation. Their peculiar characteristics, such as buffering, hydration, and force-resistance confer to these molecules additional crucial functions. GAGs can interact also with water, acting as lubricants and supporting cell migration. They are involved in the organization of collagen deposition allowing the ECM to resist high compressive forces (4, 10–13).

The dynamic structure of the extracellular matrix includes two distinct entities, the basement membranes (BM) and the interstitial matrix (IM) that are intimately interconnected. The BM is located beneath the epithelial and endothelial cells, the IM in the lamina propria, submucosa, and in muscular and serosa layers.

**Figure 1:**
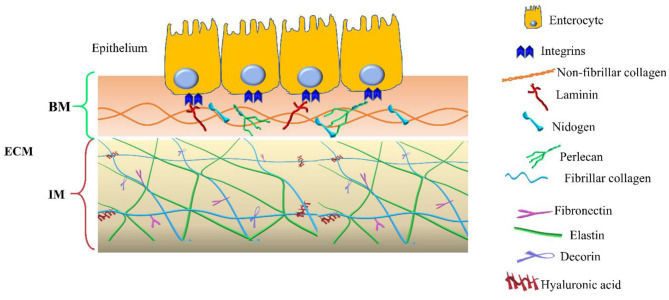
Extracellular matrix (ECM) compartments. Schematic representation of the main components of the two ECM compartments: basement membrane (BM) and interstitial matrix (IM). The legend indicates the identity of each ECM components.

In addition to the components of the basement membrane and the interstitial matrix, the transmembrane collagens and proteoglycans expressed by epithelial cells, including type XXIII collagen and syndecan-1, can also be identified (14, 15). Collagen XXIII appears to be involved in epithelial cell-to-cell contact and epithelial cell polarization. Syndecan-1 modulates epithelial cell adhesion, proliferation, and migration and stabilizes the tight junctions. The two compartments are schematized in Figure 1.

### Basement Membrane

The basement membrane is a specific 50–100 nm layer interposed between the epithelium and mesenchyme of lamina propria. The basement membrane represents a specialized form of ECM that controls cell organization and differentiation, interacting with cell surface receptors. The BM consists predominantly of collagen type IV, laminins, nidogens, and perlecan, also known as the basement membrane-specific heparan sulfate proteoglycan core protein (HSPG) or heparan sulfate proteoglycan 2 (HSPG2). Collagen and laminins can self-build more complex structures and represent the key components in BM stability, whereas nidogens and perlecan establish a complex link with laminins and collagen IV acting in the preservation of BM structural integrity (16, 17).

The BM components and their main functions are summarized in Table 2.

**Table 2:** The main components of the basement membrane and their functions.

**Molecules**	**Secreting cells**	**Functions**	**References**
Collagen type IV	Mesenchymal, enteroendocrine and epithelial cells	BM stabilization Interaction with transmembrane receptors	(18)
Laminins	Epithelial cells, Mesenchymal cells	BM assembly Modulation of epithelium homeostasis	(19–21)
Nidogens	Epithelial cells, Mesenchymal cells	BM stabilization Interaction with ECM components	(22, 23)
Perlecan	Epithelial cells, Mesenchymal cells	Cell differentiation, proliferation, adhesion and migration	(24–28)

#### Collagen Type IV

Collagen type IV is the main component of BM and interacts with integrins, the transmembrane receptors that facilitate cell–ECM adhesion, acting as mechanical links between collagen matrices and the cell cytoskeleton (18, 22, 29–31). In the bowel, the main components of BM are α1, α2, α5, and α6 chains; however, at the mucosal surface, we can also find α3 and α4 chains (18). In physiological conditions, collagen IV is synthesized mainly by mesenchymal and enteroendocrine cells, although during BM restoration, epithelial cells can temporarily carry out this function (18). Type VI and type VIII collagens are also associated with BM. Type VI is expressed throughout the crypt-villus axis and mainly located in the interface between BM and IM and directly interacting with the type IV collagen and perlecan (18, 32). Type VIII collagen is mainly expressed by endothelial cells and seems to be involved in the homeostasis of these cells and angiogenesis. It is also partly expressed by smooth muscle cells and modulates their migration (18, 33).

#### Laminins and Their Receptors

The most abundant non-collagenous adhesive glycoproteins present in BM are laminins. These molecules can bind epithelial cells and are the basis for other BM proteins exerting a key role in the onset of BM assembly. Laminins are also able to bind collagens, particularly collagen IV and XVIII (22, 29). On the basolateral surface of the intestinal epithelial cells, each laminin isoforms exerts different functions: laminin α1β1γ1 can induce differentiation, whereas laminin α5β1γ1 and laminin α3β2γ2 induce adhesion and proliferation of the epithelial cells (19–21). Laminin α5β1γ1 is expressed in the upper crypt and in the base of the villus, laminin α3β2γ2 in the villus, and laminin α1β1γ1 is exclusively present in the intestinal crypt (20). Laminins are deposited by both epithelial and mesenchymal cells (19–21).

Laminins are binding sites for cellular integrins, which show a differential expression along with human small intestinal/colon tissue. In intestinal epithelial cells, the main laminin-binding integrins are α2β1, α3β1, α7β1, and α6β4 (32). In the basal domain of intestinal cells were found α2β1 (binding laminin α1β1γ1) and α3β1 (binding laminin α3β3γ2) integrins, particularly in the crypts and on the villus, respectively. Integrin α7β1 (binding laminins α1β1γ1 and α2β1γ1) is identified in the upper part of the crypt and in the lower region of the villus axis. Integrin α6β4 (binding laminins α1β1γ1, α2β1γ1, and α3β3γ2) was detected in equally distributed from the bottom of the crypt to the top of the villus (32). Integrins represent key regulators of cell-cell and cell-ECM interactions, thus influencing growth, differentiation, as well as wound healing, and development of fibrosis. In this context, an explicative example is provided by integrin avβ6. During the pathological condition, this molecule is overexpressed and it is able to locally switch latent TGF-β in activated TGF-β, finally fueling the TGF-β-mediated fibrotic process (32).

#### Nidogens

Collagen IV can create an interconnected network with the laminins forming two sheet like networks. These can interact with the nidogens, another important BM protein, also known as entactin (23). Epithelial and mesenchymal cells express nidogens, which not only acts to stabilize the BM, but also to enhance interactions with ECM components (i.e., perlecan, laminins, and fibulin) and to mediate signal transduction through integrins (Table 2) (1, 22, 23, 34).

#### Perlecan

Perlecan is present in the BM under physiological conditions, which is a large low-density heparan sulfate proteoglycan—a molecule with GAG chains but with an independent structural domain. Perlecan is crucial for tissue development, cell proliferation, differentiation, adhesion, and migration (35, 36). The protein core contains several binding sites for collagen IV, nidogens, and integrins other than for heparin. Perlecan can interact with important growth factors, especially with the vascular endothelial growth factor (VEGF). Perlecan is synthesized by epithelial cells mainly in the basolateral surface, enhancing intestinal regeneration through the modulation of Wnt/β catenin signaling (24–28).

#### Other Components

Apart from these main constituents, the BM structure includes other fundamental molecules, such as the proteoglycan agrin, the glycoprotein fibulin, and the collagen-binding matricellular protein, also known as SPARC, Osteonectin (ON), or basement–membrane protein 40 (BM-40)—a molecule with anti-adhesion properties (23, 29, 34).

### Interstitial Matrix

The interstitial matrix is located under the BM and acts as one of the major structural layers of the lamina propria and submucosa (18). The constituents of the IM cooperate in preserving the structural and functional integrity of this compartment of the intestinal wall (Figure 1).

The main IM components and their functions are summarized in Table 3.

**Table 3:** Main components of the interstitial matrix.

**Molecules**	**Secreting cells**	**Functions**	**References**
Collagens I and III	Subepithelial myofibroblasts and fibroblasts	Regulate cell adhesion, tissue development, and homeostasis	(37–39)
Fibronectin	Fibroblasts, Epithelial cells	Regulates cell adhesion, migration, differentiation, growth, and survival	(2, 24, 40)
Elastin-tropoelastin	Fibroblasts, smooth muscle cells	Modulates intestinal tissue stretching Confers tissue elasticity	(41)
Decorin	Fibroblasts, smooth muscle cells	Mediates intestinal matrix interactions	(42)
Hyaluronan	Epithelial, smooth muscle cells and fibroblasts	Stabilizes ECM integrity	(43, 44)

#### Collagens

Collagens represent important molecules involved in the regulation of cell adhesion and tissue homeostasis (22, 45). In the gut interstitial matrix, collagen types I and III are the most representative subtypes responsible for providing tensile strength to tissues (2, 37, 46, 47). These collagens (particularly collagen I) can interact with several proteins, such as proteolytic enzymes (Metalloproteinases: MMPs), surface receptors (i.e., integrins), and other ECM molecules like fibronectin, thrombospondin, SPARC, and proteoglycans (22). In the ECM of the IM, these two collagens are synthesized and released by subepithelial mesenchymal cells (37–39).

Type V collagen is directly involved in collagen fibril assembly by interacting with type I and III collagens and forms heterotypic fibrils (fibrils composed of type I, III, and V collagens) (18, 37, 47). Types XII, XVI, and XIX of the fibril-associated collagen with interrupted triple helices (FACIT) represent the main types present in the intestine IM. These are not involved in the building of collagen fibers, but connect collagen fibrils to other ECM molecules and promote the migration of intestinal myofibroblasts (18, 37, 48).

#### Fibronectin

Fibronectin is a glycoprotein present as an insoluble form into the IM (2, 4, 22). It interacts with several ECM molecules such as collagens (including types II, III), heparin, tenascin-C, and cell surface receptors of the integrin superfamily. Fibronectin is involved in several cellular activities taking place in connection with the ECM, such as cell adhesion, growth, migration, differentiation, and survival (2, 11, 22, 49, 50). Fibronectin plays an important role in the homeostasis of the barrier function of the intestinal mucosa and is constantly exposed to luminal bacteria and toxins. During a mucosal injury, fibronectin participates in the restoration of epithelial integrity. Fibroblasts as well as epithelial cells are the main producers of the intestinal fibronectin (2, 24, 40).

#### Elastin

Elastin is secreted as tropoelastin and represents another important component of the interstitial matrix that confers elasticity and resilience to intestinal tissue. It is responsible for the tissue's ability to recoil following repeated expansion and contraction stretching; however, its amazing elasticity is limited by the intimate association with collagen fibrils (51, 52). Tropoelastin is produced by fibroblasts, smooth muscle cells, and endothelial cells before it is processed to elastin by cleavage of its signal peptide (41).

#### Decorin

Chondroitin sulfate proteoglycan decorin and the glycosaminoglycan hyaluronan are also present in the IM (18). These molecules can interact with several ECM proteins (i.e., collagens), cytokines (i.e., tumor necrosis factor-alpha, TNF-α), and growth factors such as transforming growth factor-β (TGF-β) and platelet-derived growth factor (PDGF) (53–57). Decorin selectively interacts with different molecules: the isoforms of TGFβ, PDGF, vascular endothelial growth factor receptor-2 (VEGFR-2), epidermal growth factor receptor (EGFR), connective tissue growth factor (CTGF), thrombospondin, collagens, and fibronectin. Decorin is mainly expressed by fibroblasts, smooth muscle cells, and macrophages (42).

#### Hyaluronan

Hyaluronan (HA) is an abundant ECM component produced by epithelial cells, smooth muscle cells, and fibroblasts (43, 44). It exerts an important role in regulating the hydration of tissues, as well as affects cell adhesion, migration, and mitosis. It also acts as an anti-angiogenic and anti-inflammatory factor. Almost all of these effects are mediated by the hyaluronan receptor CD44, which is expressed by stromal and immune cells (58). Hyaluronan also acts in the homeostasis of intestinal stem cells (ISC) through the interaction with constituents of the extracellular matrix contributing to stabilizing its integrity. It is mainly expressed on the plasma membrane of ISCs and its structure contains binding sites for the Toll-like receptors activated in the response to commensal and pathogenic bacteria (59, 60). The hyaluronan level of polymerization indicates matrix integrity. In contrast, elevated hyaluronan' fragments were found to be associated with inflamed tissue in IBD inducing leukocyte infiltration into the intestine and innate immune activation (44, 61). Fragments of hyaluronan can promote wound healing, but also fibrosis, by inducing fibroblast proliferation and myofibroblast differentiation (59, 61).

## ECM Is a Dynamic Structure: Cellular and Molecular Functions

The ECM does not represent a static structure, but a dynamic tissue component that constantly undergoes continuous remodeling (10, 11, 18, 22, 62). The homeostasis of healthy tissue is guaranteed by a continuous and balanced deposition, degradation, and modification of the ECM and an imbalance in this equilibrium can lead to pathological conditions (63–65). A large number of molecules and growth factors orchestrate this delicate process by regulating ECM amount, composition, and structure (10, 11, 18, 62). Intestinal fibrosis, characterized by an abnormal deposition of ECM, represents the main chronic complication of inflammatory bowel disease (IBD), chronic relapsing intestinal disorders including Crohn's disease, which can affect both the small and large intestine, and ulcerative colitis, which only affects the large intestine (5, 10).

### ECM-Producing Cells

The intestinal mucosa consists of three distinct portions: a single layer of epithelial cells, a connective tissue that keeps the epithelium (the lamina propria) in place, and a small layer of smooth muscle, called muscularis mucosae, which separates it from the underlying muscle layers. The various components of the ECM are produced by different types of intestinal cells. While the components of the basement membrane are produced by epithelial cells, those of the interstitial matrix are mainly produced by mesenchymal cells represented by fibroblasts, myofibroblasts, and smooth muscle cells. Endothelial cells, pericytes, and stellate cells also contribute to the release of ECM components. ECM-producing cells act synergistically and are under the control of numerous biological mediators. The ECM-producing cells and their main markers are summarized in Table 4.

**Table 4:** Intestinal ECM-producing cell types.

**Cell type**	**Main positive markers**	**References**
Epithelial cells	E-cadherin, cytokeratins, CD326	(5, 66–68)
Fibroblasts	Vimentin, CD90, N-cadherin (high), prolyl 4-hydroxylase	(69, 70)
Myofibroblasts		
- Subepithelial myofibroblasts - Interstitial cells of Cajal	Vimentin, α-SMA, cadherin-11, epimorphin Vimentin, c-Kit receptor, anoctamin-	(5, 71, 72)
Smooth muscle cells	α-SMA, desmin, smoothelin, HDAC-8	(73, 74)
Endothelial cells	CD31, vWF, VE-cadherin, N-cadherin (low), vimentin (low)	(67, 75)
Pericytes	NG-2, α-SMA, desmin (low), MCSP, RGS5, PDGFRB, CD11b, CD80, CD86, CD13, CD90, ANG I, and II, ET-1	(5, 67, 76)
Stellate cells	Vitamin A, GFAP, desmin	(5, 67, 77)

#### Epithelial Cells

At least five cell types can be found in the intestinal mucosal epithelium: enterocytes, goblet cells, Paneth cells, enteroendocrine cells, and stem cells. They are found both in the intestinal glands and on the surface of the villi. The enterocytes are specialized in the absorption of water, electrolytes, and nutrients; the goblet cells secrete different types of mucins; the Paneth cells help to maintain mucosal immunity by secreting antimicrobial substances; the enteroendocrine cells produce various paracrine and endocrine hormones; the stem cells guarantee the physiological renewal of all of the above types of epithelial cells or when they are damaged. In chronic inflammatory bowel diseases, epithelial cells can undergo a well-known process of epithelial-to-mesenchymal phenotypic transformation, becoming one of the main sources of activated myofibroblasts, and thereby being directly involved in the processes of tissue repair and fibrogenesis (5, 66, 67).

#### Fibroblasts

Fibroblasts are a heterogeneous population of cells located in the interstitium of all normal tissues and organs where they are central in maintaining structural integrity, healing, and regeneration, by regulating matrix homeostasis. Fibroblasts are directly involved in the pathogenesis of intestinal fibrosis (69).

#### Myofibroblasts

Myofibroblasts are highly contractile cells that exhibit a “hybrid” phenotype between fibroblasts and smooth muscle cells (SMCs) and, when activated, synthesize high levels of ECM, particularly collagen, glycosaminoglycans, tenascin-C, and fibronectin (5, 71, 78). Besides roles in tissue growth and differentiation, myofibroblasts are central to wound healing and fibrosis (5, 67). Two types of myofibroblasts are present in the intestinal mucosa in physiological conditions, the intestinal sub-epithelial myofibroblasts (SEMFs) and the interstitial cells of Cajal (ICC) (72, 79). Sub-epithelial myofibroblasts are located at the base of the intestinal crypts in the lamina propria, form a three-dimensional network, and are in connection with each other, but also maintain connections with epithelial cells. Myofibroblastic cells contain smooth muscle cytoskeletal markers (in particular α-smooth muscle actin: α-SMA) together with three filaments (vimentin, desmin, or myosin), with variable expression depending on tissue, species and environmental factors (72, 79). Activated myofibroblasts play the main role in the development of intestinal fibrosis (5, 67). Interstitial cells of Cajal are located in the submucosa and muscularis propria in association with the smooth muscle layer (80, 81). Interstitial cells of Cajal are pacemaker cells, which regulate intestinal smooth muscle motility.

#### Smooth Muscle Cells

Smooth muscle cells (SMCs) are one of the three interrelated cell phenotypes into which intestinal mesenchymal cells can differentiate (the other two being fibroblasts and myofibroblasts) (73). In chronic inflammation, SMCs can trans-differentiate into myofibroblasts, suggesting that a dynamic equilibrium thus exists between the myofibroblast and SMC phenotype (5, 67, 73).

#### Endothelial Cells

Endothelial cells (ECs) are the major constituent of the microvasculature that line blood and lymphatic vessels. Normally, ECs provide an anti-adhesive and selectively permeable exchange barrier. Endothelial cells, by continual adjustments in structure and functions, coordinate vascular supply, immune cell migration, and regulation of the tissue environment. Inflammation induces changes in the endothelium of the intestinal vasculature in response to the cytokines, chemokines, and growth factors released by immune and non-immune cells, leading to decreased endothelial barrier function, adhesion molecule expression, leukocyte extravasation, and increased coagulation and angiogenesis (82). Intestinal inflammation, especially in chronic inflammatory bowel diseases, can induce endothelial cells to undergo endothelial-to-mesenchymal phenotypic transformation, becoming a further source of activated myofibroblasts, and thereby being directly involved in the processes of tissue repair and fibrogenesis (67, 75).

#### Pericytes

Pericytes are derived from undifferentiated mesenchymal cells and surround capillary and small blood vessel endothelial cells (76, 83). They reside at the interface between the endothelium and interstitium. Pericytes display an intermediate phenotype between vascular SMCs and fibroblasts. Pericytes control endothelial cell differentiation, endothelial signaling, angiogenesis, and ECM deposition (5, 67, 76). They represent a useful reserve of myofibroblasts during tissue repair and inflammation-associated fibrosis.

#### Stellate Cells

Stellate cells are mesenchymal cell precursors that contribute to retinoic acid metabolism, which impacts fibrosis and when activated, may differentiate into myofibroblasts (84, 85); however, limited information is available for intestinal stellate cells, although, in chronic inflammatory bowel diseases, we know that they differentiate into myofibroblasts faster than those from normal mucosa and proliferate faster, and produce collagen earlier and at higher levels (5, 67).

### Enzymes Degrading ECM Proteins and Their Inhibitors

One of the main enzymes that can degrade ECM components is metzincins.

**Figure 2:**
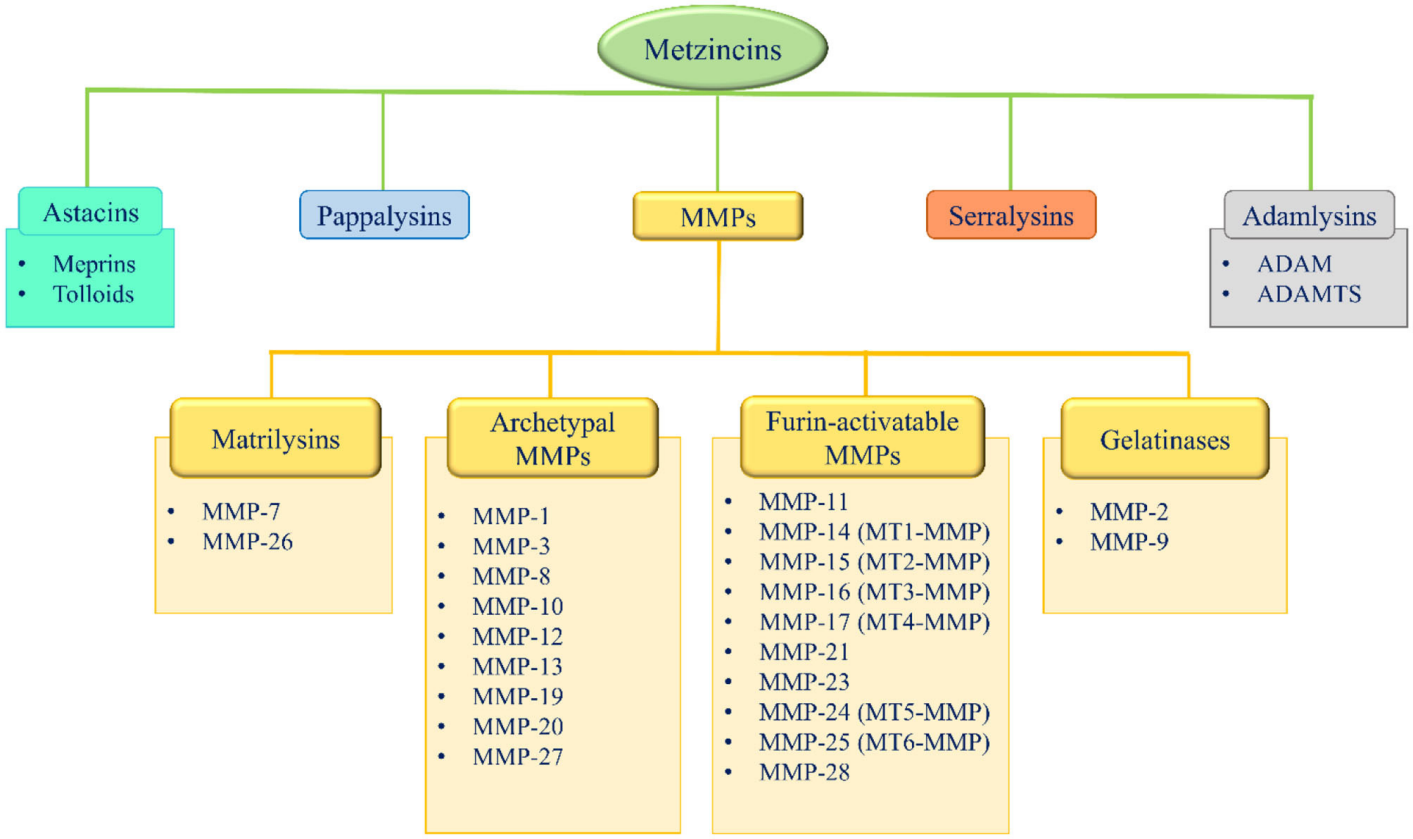
Metzincins superfamily members and classification of MMPs based on their domain arrangement. MMP, metalloproteinase; MT-MMP, membrane-type MMP; ADAM, a desintegrin and metalloproteinase; ADAMTS, a-desintegrin and metalloproteinase with thrombospondin motif.

Metzincins represent a superfamily of zinc-dependent endopeptidases present in the ECM and are classified in: astacins, pappalysins, MMPs, serralysins, and adamalysins, including a-desintegrin and metalloproteinase (ADAMs) and a-desintegrin and metalloproteinase with thrombospondin motif (ADAMTS) (86–89) (Figure 2). From 1962, 23 MMPs, 21 ADAMs, and 19 secreted ADAMTS are identified in humans (87, 89). The main function of these proteases is the degradation of ECM proteins, but they also exert an important role in crucial physio-pathological processes such as enzymatic activities, protease inhibition, protein synthesis inhibition, cell proliferation, migration and apoptosis, inflammation, wound healing, fibrosis, angiogenesis, and carcinogenesis (86–110).

Meprins are members of astacins, existing in two isoforms (α and β), that play a key role in connective tissue homeostasis, especially in cell migration, differentiation, and proliferation (111, 112). These enzymes can cleave the N- and C-terminal pre-domains of procollagens I and III, an essential step for the correct assembly of collagen fibril (113, 114). Meprins are involved in collagen IV, nidogens, and fibronectin other than SPARC and fibulin cleavage (115–117). In the bowel, meprins are also crucial in the preservation of intestinal barrier functions. Particularly, meprin β is located on the apical side of the epithelial cells and prevents bacteria attachment and invasion; however, during pathological processes such as inflammation, the expression of fibrosis and cancer meprins increased (111, 118).

ADAMs are membrane-anchored metalloproteinases that exert proteolytic activity inhibiting the metalloproteinase domain. Likewise, they play a key role in cell–cell interactions by connecting heparan sulfate proteoglycans with integrin proteins (11, 119–122).

Beside ADAMs, other components of the adamalysins family are the ADAMTS. In these molecules, near to a disintegrin domain, there is a thrombospondin type 1-like repeats sequence that confers to ADAMTS the ability to bind ECM proteins (11, 123).

MMPs are produced in a precursor form (PRO-MMPs) and remain in a status of low activity. They are confined in specific areas, while their expression increases during processes of remodeling, repair, or in the presence of chronic inflammatory or neoplastic diseases in many organs, including the intestine (8, 11, 92–96). The presence of MMPs is generally low in normal or uninflamed tissues. The expression, secretion, activation, and activity of MMPs are tightly controlled (90). The activity of MMPs is regulated on several levels, including transcription, translation, secretion, activation by cleavage of the pro-domain, and inhibition by the endogenous TIMPs (61). MMPs play a crucial role in the physiological turnover of the ECM bowel.

#### Matrix Metalloproteinases

A significant contribution to intestinal ECM remodeling is ascribable to MMPs, which can be classified mainly by two different criteria: based on their domain organization or their substrate preferences (86–89, 124–126).

According to their domain arrangement, MMPs are classified as matrilysins, archetypal MMPs, furin-activatable MMPs, and gelatinases (Figure 2) (86–89, 124–126).

According to their substrate specificity, MMPs are classified as collagenases (MMP-1, −8, −13, and −18, this latter is not expressed in mammals, but only in Xenopus), gelatinases (MMP-2 and 9), stromelysins (MMP-3, −10, and −11), metrilysins (MMP-7 and −26), membrane-type MMPs (MT-MMPs) (MT1-MMP, MT2-MMP, MT3-MMP, MT4-MMP, MT5-MMP, and MT6-MMP) and other MMPs (MMP-12, −19, −20, −21, −23, −27, and −28) (86–89, 113–115). Specific substrates have been identified for the majority of the MMPs (Table 5).

**Table 5:** Classification of MMPs ECM degrading enzymes and their main substrates.

**Class names**	**Molecular names**	**Preferential substrates**	**References**
Collagenases	MMP-1 MMP-8 MMP-13 MMP-18	Collagens (I, II, III, VII, VIII, X, XI), gelatin, fibronectin, vitronectin, laminins, entactin, tenascin, aggrecan. Collagens (I, II, III), aggrecan. Collagens (I, II, III, IV, VI, IX, X, XIV), gelatin, fibronectin, aggrecan, SPARC, perlecan. Collagen I.	(11, 86–89, 113–115)
Gelatinases	MMP-2 MMP-9	Collagens (I, II, III, IV, V, VII, X, XI) gelatin, elastin, fibronectin, vitronectin, laminins, entactin, tenascin, SPARC, aggrecan, decorin. Collagens (IV, V, X, XIV), gelatin, elastin, vitronectin, laminins, entactin, tenascin, SPARC, aggrecan, decorin.	(11, 86–89, 113–115)
Stromelysins	MMP-3 MMP-10 MMP-11	Collagens (III, IV, IX, XI), gelatin, elastin, fibronectin, vitronectin, laminins, entactin, tenascin, SPARC, aggrecan, decorin, perlecan. Collagens (III, IV, V), gelatin, elastin, fibronectin, aggrecan. Collagen IV, gelatin, fibronectin, laminins.	(11, 86–89, 113–115)
Matrilysins	MMP-7 MMP-26	Collagens (I, IV), gelatin, elastin, fibronectin, vitronectin, laminins, entactin, tenascin, SPARC, aggrecan, decorin. Collagen IV, gelatin, fibronectin, vitronectin.	(11, 86–89, 113–115)
Membrane type (MT)	MMP-14 (MT1-MMP) MMP-15 (MT2-MMP) MMP-16 (MT3-MMP) MMP-24 (MT4-MMP)	Collagens (I, II, III), gelatin, fibronectin, tenascin, vitronectin, laminins, entactin, aggrecan, perlecan. Fibronectin, tenascin, entactin, laminins. Collagen III, gelatin, fibronectin, vitronectin, laminin. Fibronectin, gelatin, chondroitin sulfate proteoglycan, dermatan sulfate proteoglycan.	(11, 86–89, 113–115) (11, 86–89, 113–115)

Several ECM components undergo limited proteolysis generating bioactive fragments called matricryptins, which regulate many physiological and pathological processes through their binding to cell surface receptors. The major physiopathological processes regulated by matricryptins include enzymatic activities, protease inhibition, protein synthesis inhibition, cell proliferation, migration and apoptosis, inflammation, wound healing, fibrosis, angiogenesis, and carcinogenesis (91).

In addition to a crucial role in the ECM components degradation, MMPs have a vast range of extracellular, pericellular, and intracellular substrates (90). At the mucosal surface level, antibacterial molecules, such as the membrane-bound mucin-1 (MUC1) and defensins, can be modified by MMPs, leading to the alteration of host–bacterial interaction. Within the epithelial layer, MMPs can degrade intercellular junction molecules (e.g., cadherins, occludins, and claudins) and intracellular structural proteins (e.g., actins), leading to the alteration of the cell shapes and the barrier function. The degradation of ECM components (e.g., collagens) may release several chemotactic and angiogenic (e.g., endostatin) fragments. MMPs proteolytically activate or degrade a variety of non-matrix substrates, including chemokines, cytokines, adhesion molecules, growth factors, and survival molecules; therefore, MMPs are increasingly recognized as critical players in the intestinal inflammatory response, tissue repair, fibrogenesis, and carcinogenesis (90, 92–110).

The activity of the ECM degrading enzymes is balanced by specific inhibitors represented by the tissue inhibitors of metalloproteinases (TIMPs).

In the bowel, MMPs and TIMPs are mostly investigated in the mucosa but are also present in the submucosa and muscolaris propria. The healthy epithelium expresses a wide range of MMPs and TIMPs, but in the injured epithelium, MMP-7 and MMP-10 are more pronounced (90). TIMP-3 is mainly associated with a healthy intestine and is reduced in the inflamed intestine. Stromal cells such as fibroblasts also express many MMPs and TIMPs, but fibroblasts containing MMP-1, −3, −8, and −9 have been associated with inflamed intestine (90). Immune cells also contribute to MMP and TIMP expression in the mucosal and submucosal layers (90).

#### Metalloproteinases Inhibitors

Metalloproteinases activity is fine regulated by activation of synthetic and endogenous (non-specific and specific) inhibitors (86–89, 124–126).

Synthetic inhibitors including hydroxamate-based inhibitors, non-hydroxamate-based inhibitors, catalytic domain inhibitors, allosteric and exosite inhibitors, and antibody-based inhibitors (124, 125).

Among the endogenous non-specific inhibitors there are α-macroglobulin, tissue factor pathway inhibitor (TFPI), membrane-bound β-amyloid precursor protein, C-terminal proteinases enhancer protein, reversion-inducing cysteine-rich protein with Kasal domain motifs (RECK), and GPI-anchored glycoprotein; however, the main regulators of MMPs other than the adamalysins activity, are the TIMPs (124, 125).

The tissue inhibitors of metalloproteinases family are composed of four members (TIMP-1, −2, −3, and −4) and an important parameter in the control of their function is their localization. While TIMP-1, −2, and −4 are present in soluble form, TIMP-3 is sequestered in the ECM through the interaction with HS and GAGs (11, 126–133). Although TIMPs globally inhibit all the MMPs, each TIMP showed a preferential substrate (124–133) (Table 6).

**Table 6:** MMPs inhibitors and their preferential substrates.

**Inhibitory enzymes**	**Preferential substrate**					**References**
**TIMPs**	**PRO-MMPs**	**MMPs**	**MT-MMPs**	**ADAMs**	**ADAMTS**	
TIMP-1	PRO-MMP-9	MMP-1 MMP-2 MMP-3 MMP-9 MMP-19	MT1-MMP MT3-MMP MT5-MMP	ADAM-10	–	(11, 126–133)
TIMP-2	PRO-MMP-2	MMP-2	–	–	–	(11, 126–133)
TIMP-3	PRO-MMP-9 PRO-MMP-2	MMP-1 MMP-2 MMP-3 MMP-9 MMP-13	–	ADAM-12 ADAM-17	ADAMTS-1 ADAMTS-4 ADAMTS-5	(126–133)
TIMP-4	PRO-MMP-2	MMP-2	MT1-MMP		–	(124–133)

### Growth Factors Controlling ECM Deposition and Remodeling

**Figure 3:**
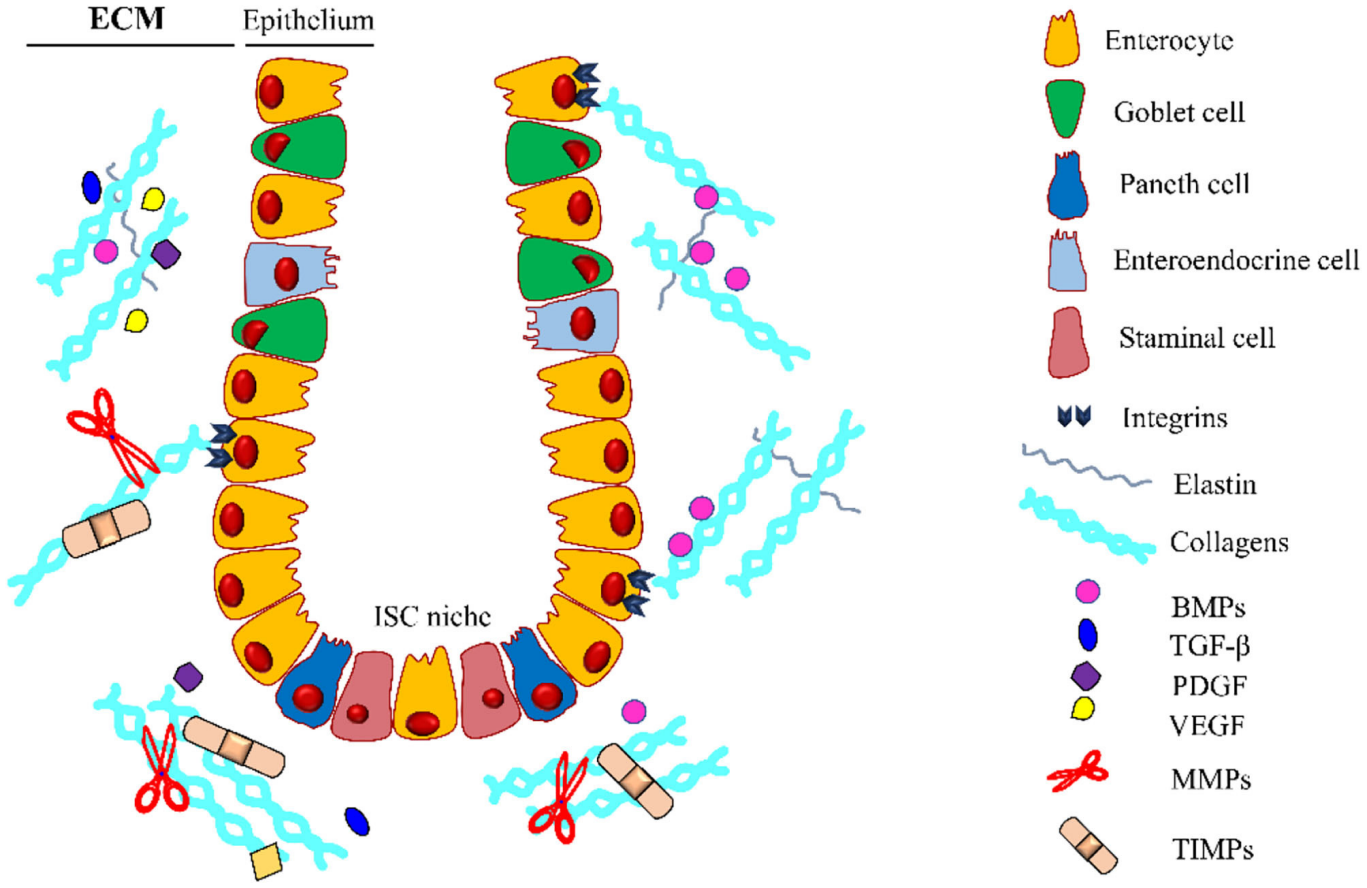
Intestinal crypt microenvironment. Schematic representation of an intestinal crypt with the cells and the molecules involved in its homeostasis. At the base of the invagination, there is the ISC niche, essential for supplying the staminal cell pool involved in the physiological self-renewal of the epithelium. Growth factors (GFs) are entrapped in the extracellular matrix (ECM), which is constantly remodeled by the coordinated activity of ECM-producing cells, metalloproteinases (MMPs), and tissue inhibitors of metalloproteinases (TIMPs). Along the crypt, there is a gradient for some GFs (i.e., BMPs) regulating the differentiation and proliferation of the intestinal stem cells (ISCs). BMPs, Bone Morphogenetic Proteins; TGF-β, Transforming Growth Factor-β; PDGF, Platelet-Derived Growth Factor; VEGF, Vascular Endothelial Growth Factor. The legend indicates the crypt components.

The extracellular matrix can act as a reservoir for growth factors released or activated upon MMPs mediated proteolysis, once released, these molecules affect cell recruitment and function, inducing ECM deposition and restoring its integrity. These growth factors are directly responsible for the maintenance of the tissue and repair of the intestinal epithelium (Figure 3).

The main growth factors controlling ECM deposition are represented by TGF-β1, activins, CTGF, Fibroblast growth factor (FGF), PDGF, epidermal growth factor (EGF), insulin-like growth factor (IGF)-I and II, VEGF, bone morphogenetic proteins (BMPs), and hepatocyte growth factor (HGF).

Transforming growth factor-β is the crucial growth factor involved in the biological regulation of ECM protein synthesis. The main producers of TGF-β are represented by epithelial cells, immune cells, and fibroblasts, which are highly expressed in the lamina propria of a healthy bowel (134, 135). TGF-β exists in three different isoforms (TGF-β 1, 2, and 3) secreted in a latent state (LTGF-β) and often bound to a second protein, the latent TGF-β binding protein (LTBP). This complex facilitates the secretion and direction of ECM components, mainly fibronectins and fibrillins (134–143).

TGF-β interacts with the small mother against decapentaplegic (Smad) family proteins that induce its nuclear translocation, regulating its signaling, and the formation of transcriptionally active complexes (29, 144–149). During physiological intestinal ECM turnover, TGF-β plays a key role in the regulation of the expression of the collagen and the laminins proteins (22). Additionally, TGF-β1 through the interaction with Smad3 can induce procollagen I and III depositions by intestinal fibroblasts (143, 147). Besides Smads downstream pathways, TGF-β1 can also modulate, in a Smad/independent manner, other signal transduction pathways, such as ERK/cJUN/p38MAP kinases and the phosphoinositide-3 kinase (PI3-K) and its downstream target Akt, also known as protein kinase B (PKB), as well as members of the JAK and STAT protein family (143–147). It is not yet fully understood which of these transduction pathways mainly modulate the anti-inflammatory effect and which the pro-fibrotic effect of TGF- β (143–147); however, TGF-β exerts several crucial functions in the bowel. TGF-β plays an important role in the crosstalk between the host immune cells and the gut microbiota, both in the small intestine (regulating the complex microbiota including *Lactobacillus* sp., *Streptococcus* sp. *Clostridium* sp., *and Escherichia coli)* and in the colon (rich in *Clostridium* sp. *and Bacteroides species*). After an epithelial injury or an inflammation, intestinal epithelial cells increase the production of the TGF-β. In turn, the microbiota release molecules (butyrate, acetate, and propionate) to enhance the production of TGF-β by epithelial cells, regulating the immune response (150–154). Although TGF-β shows an anti-inflammatory effect during acute intestinal inflammation, in a chronic phase of the disease, it induces a pro-fibrotic effect. In this context, TGF-β is a key regulator of intestinal fibrosis (5, 63, 67).

Other members of TGF-β superfamily are the BMPs and activins. BMPs play an important role during homeostasis by controlling cellular differentiation, proliferation, and apoptosis (155–157). In the intestine, the ECM regulates the position, the timing, and the intensity of the BMPs activity (158). The BMPs are sequestered in ECM, mainly interacting with fibrillin and collagen IV (159). BMPs promote epithelial differentiation in the crypts and inhibit the expansion of the stem cells pool (160, 161). A peculiar gradient of BMPs is reported in the intestine, degrading from the villus toward the base of the crypt (161). Endogenous BMP antagonists exist, such as Gremlin1, Gremlin2, and Chordin, secreted by the intestinal subepithelial myofibroblasts and smooth muscle cells (162). Furthermore, BMP2 and 7 are inhibited by angiopoietin-like-protein 2 expressed by subepithelial myofibroblasts to maintain ISC homeostasis (163, 164). Activins represent an important player in the regulation of the intestinal epithelial cell functions. Activin A and its receptors modulate epithelial cells migration and proliferation exerting a positive role during the intestinal inflammation and wound healing processes (164). Activins activate Smad transcription factors and the MAP kinase signaling pathways (165).

The connective tissue growth factor is a downstream mediator of TGF-β. It is co-expressed with TGF-β and stimulates cell proliferation and ECM synthesis. Its expression is controlled by TGF-β in a Smad-dependent manner. In IBD, the activation of the TGF-β pathway induces an increased expression of CTGF that leads to abnormal local deposition of ECM components and intestinal fibrosis. In addition to TGF-β, other modulators of CTGF expression include VEGF, TNF-α, and ROS (166, 167).

Fibroblast growth factor (FGF) is intimately associated with ECM, especially with HSPG present in the BM (29). The interaction with its receptor (FGFR), expressed on fibroblasts and epithelial cells of the intestinal crypt, can regulate the synthesis of specific ECM components such as collagens, laminins, and fibronectin (22, 168–170).

Another molecule associated with ECM proteins is represented by PDGF. This factor is mainly expressed by the epithelial cells, but also endothelial cells, fibroblasts, and smooth muscle cells it is a positive regulator of stromal cell proliferation (171). Plated-derived growth factor-AA and its receptor (PDGFR), are crucial for the proper structure of the intestinal mucosa. Knockout mice for PDGF-AA or PGFR-A showed a reduction in the enterocyte turnover, and the subepithelial mesenchymal cluster aggregation, as well as a decrease in villus formation (172). The activity of PDGF and its diffusion in the tissue interstitium is regulated by the binding with ECM proteins, decorin, HS, and SPARC, that sequester PDGF in extracellular space in an inactive form and inhibit its action (36, 140, 173).

Similarly, the VEGF is secreted by the enterocytes, endothelium, and muscularis layer and remains spatially confined through HS interaction. Once ECM is remodeled, VEGF is released and controls cell proliferation, migration, and differentiation, and angiogenesis (174, 175).

Insulin-like growth factor-I and II and their respective receptors are expressed in the intestine and interact principally with fibroblasts, epithelial, and endothelial cells and regulate collagen deposition. IGF-1 enhances myofibroblast migration and increases intestinal SMC and myofibroblasts (36, 168–178). Epidermal growth factor is the prototype member of a family comprising different peptides with a similar primary structure that binds to a family of EGF receptors. It regulates transcription, translation, cell architecture, and cell proliferation. EGF can be isolated from the intestine and its receptors are located on monocytes and myofibroblasts. EGF stimulates fibroblast proliferation and ECM production and regulates human colonic fibroblast and myofibroblast migration (179–181).

Hepatocyte growth factor (HGF) and BMP-7 are natural inhibitors of the TGF-β/Smad pathway (182, 183). HGF exerts several biological activities on myofibroblasts, including the inhibition of growth and ECM deposition and the increase of MMP expression (184, 185). BMP-7 downregulates α-SMA and phosphorylated Smad2/3 (183).

### Extrinsic Regulation of ECM: Metabolic and Mechanical Control

ECM organization and functions are under the control of metabolism and metabolic regulators (186–188). The main cellular metabolic sensor, AMP-activated protein kinase (AMPK) joins the cell energetic status with the cell-to-ECM adhesion and the ECM deposition (186). In fact, AMPK has been reported to inhibiting the expression of the integrin-binding proteins, tensin 1 and tensin 3 in several systems. Moreover, AMPK is able to phosphorylate proteins of cell-to-cell adhesion, such as claudin 1, claudin 4, and cingulin, enhancing the epithelial barrier and cell polarity (186–188). Activation of AMPK induces the suppression of the TGF-β1 signaling and prevents abnormal ECM remodeling with excessive collagen synthesis deposition and tissue fibrosis (189). Another important metabolic mediator involved in ECM function is the yes-associated protein 1/transcriptional coactivator with PDZ-binding motif (YAP/TAZ) pathway (190, 191). YAP/TAZ signaling integrates the energetic status to adhesion on ECM and activation of YAP facilitates the cell contact to a stiff ECM. Interestingly, YAP is also reported to be involved in tissue regeneration and ISC activation. Upon injuring conditions, ECM releases factors binding the integrin β1 and activating focal adhesion kinase (FAK)/Src pathway. Finally, YAP/TAZ is triggered and leads to stem cell activation and tissue repair (190–192). Furthermore, key regulators of lipids, and carbohydrates metabolisms, such as PPAR-γ, mTOR, and the adipokine leptin, are able to highly modulate ECM functions (134, 149, 193, 194). Indeed, these molecules influence ECM behavior acting on ECM-producing cells. Particularly, PPAR-γ leads to a decrease of ECM proteins deposition exerting an anti-fibrotic effect, while mTOR and leptin activate myofibroblasts resulting in abnormal deposition of ECM, and the onset of the fibrotic process (134, 149, 193, 194).

The intestinal mucosa is constantly subject to mechanical stimuli that can activate molecular signals on the epithelial cells and leads to alterations of the epithelium biology. Mainly two forms of mechanical forces act on the intestinal wall: contractile activities (i.e., peristalsis) and shear stress (due to the presence of food, gas, and fluids) (192, 195, 196). These forces heavily impact bowel morphogenesis. An interesting study conducted on rat's small intestine revealed that ECM fibers are organized as two interwoven arrays running diagonally around the crypt wall: one set clockwise and the other one set counterclockwise and oriented at a range of angle ± 30–50° (192, 195–197). This spatial arrangement provides mucosa with the right flexibility to fit stretch forces (192, 195–197). The ECM conveys this mechanical information to cells through an integrin network, activates focal adhesion kinase (FAK) and the extracellular signal-regulated kinase (ERK) signaling, finally leading to cell proliferation. Shear stress and mechanical stretch were also reported to induce cyclo-oxygenase-2 and other pro-inflammatory mediators [interleukin (IL)-6, IL-8, and monocyte chemoattractant protein 1] (198).

Furthermore, a high impact on ECM deposition is provided by changes in the tensile forces and elasticity of the intestinal wall. Indeed, a reduction of these parameters leads to alterations of ECM physical properties, such as ECM stiffness. Tissue stiffness is established by ECM composition and contraction, as well as by ECM-producing cells (fibroblast, myofibroblasts, and smooth muscle cells) contractility, under the control of the master regulator TGF-β (63).

## Extracellular Matrix and Intestinal Stem Cell Niche

The ECM can undergo peculiar compartmentalization, creating specialized tridimensional invagination in both the small and large intestines, known as the crypt, the hub for stem cell pool, allowing fast turnover (4–5 days) of the epithelium (185, 199–201). At the base of the crypt reside the intestinal stem cells (ISCs), a heterogeneous group of cells nourished by a surrounding cell niche. This niche, known as the intestinal stem cells niche, acts in physiological epithelial tissue regeneration, self-renewal, and differentiation other than in the repair of intestinal mucosal epithelium after injury (199–201). Several studies highlighted that ISCs are regulated by many signaling pathways, mainly by Wnt, bone morphogenic proteins (BMPs), and Notch signaling, involved both in physiologic and pathologic conditions (202–205). Particularly, in ISCs, Wnt is involved in the regulation of intestinal epithelial renewal in physiological conditions, BMPs acts in the intestinal development and the epithelial homeostasis, and Notch ensures that the crypt compartment remains in an undifferentiated and proliferative state during gut development (206–210). The ISC niche can be described as a combination of physical and cellular niches (2). The physical niche includes the complex network of ECM proteins (i.e., fibronectins and collagens) that provides a crucial scaffold to maintain the correct architecture of the intestine (1, 211, 212) (Figure 3). The cellular niche is the stromal microenvironment composed of all resident cells dipped in the ECM. These cells included myofibroblasts, fibroblasts, smooth muscle cells, endothelial cells, and pericytes that cooperated to secrete several growth factors and matrix components that are crucial for maintaining the homeostasis of ISCs by regulating their proliferation and differentiation (73, 200, 213).

## Conclusions

The intestinal ECM is a complex structure consisting of a mixture of proteins, the glycosaminoglycan hyaluronan and proteoglycans that interact to make a scaffold for the resident cells. The core matrisome of the ECM consists of more than 300 proteins, each of them with unique biochemical properties conferring exclusive biological functions (5, 10). These proteins give rise to distinct ECM compartments: the basement membrane (BM) and the interstitial matrix (IM). The structural proteins (collagens) in ECM provides a scaffold for the tissue and mainly endures the tensile strength, limiting the stretching of the organ. Glycoproteins (laminins, elastins, fibronectin, and nidogens) are involved in the ECM assembly and cell-to-ECM interaction by binding cell receptors as integrins. The PGs (perlecan and decorin) and the GAG (hyaluronan) retain water in the tissue, conferring hydration, and entrap growth factors that influence cell behavior. The ECM contributes to establishing a selective barrier to the external environment (5, 10). The extracellular matrix is a dynamic structure frequently remodeled by the orchestrated activity of degrading enzymes and their inhibitors (10, 11, 90, 92). During this remodeling, ECM releases growth factors affecting and regulating resident cell function and finally ECM deposition. This cell-to-ECM crosstalk is pivotal for the physiological homeostasis of the intestine, but the plethora of mechanisms controlling this reciprocal communication are not yet fully known. In this context, the extracellular vesicles (EVs), which are complex phospholipidic structures (20 up to 1,000 nm) released by prokaryotic and eukaryotic cells in the biological fluids, appear to play an important role. During their biogenesis, EVs acquire the molecular legacy of the donor cells and can shuttle biological information far away from the cells (214–219). For this reason, EVs are recognized as a new means of intercellular and ECM-cell communication, cooperating in the physiological and pathological regulation in many organs (214, 220–223). In this context, intestinal epithelial cells (IECs) produce EVs that can regulate the TGF-β pathway, which is pivotal for intestinal homeostasis (224, 225). EVs released by IECs contain TGF-β that can bind its receptor on target cells, but also carry the integrin αvβ6 that induces the activation of the LTGF-β in ECM, releasing the active factor. Metalloproteinases have also been described to be shuttled by EVs exerting their activity, such as MMP-2, −3, −9, −13, and −14 as well as TIMPs (TIMP-2) (225–228).

All that we have described and shown in this ECM overview is only a piece of a bigger and more complex puzzle. The intestinal homeostasis is correctly prompted by a multifaced interaction between different systems. One of the main interlocutors is undoubtedly the intestinal microbiota, the large mass of symbiotic commensal microorganisms presents in the gut lumen. The presence of microorganisms strongly regulates the health and physiology of the intestine, since commensal bacteria, especially those attached to the mucous surface, contribute together with the intestinal epithelium to the integrity of the important intestinal barrier function. The intestinal microbiota continuously interacts with the local immune system, creating a mutually beneficial balance between them. Interestingly, this interaction between the microbiota-gut-immune system is partially modulated by EVs (219). Gut microbiota physiologically produces numerous metabolites, such as short-chain fatty acids and Krebs cycle anions which have significant effects on the homeostasis of intestinal epithelial cells, but also of ECM-producing cells and immune cells. Microbiota produce several proteases that can alter the barrier functions of the intestinal mucosa.

Acute intestinal damage activates the acute inflammatory response which, in turn, initiates the well-known mucosal healing processes mediated by the ECM remodeling. Unfortunately, the factors and/or mechanisms that lead to an abnormal ECM turnover in the course of chronic intestinal inflammation, which is also responsible for complications that can develop over time, such as intestinal fibrosis and intestinal cancer, are not yet known. It is now established that disturbance of the fine-tuned regulation of ECM remodeling is the cornerstone of several gut diseases, including chronic inflammation, fibrosis, and cancer (64, 67, 229, 230). Although huge steps in the knowledge of the ECM have been made over the last decade, further investigations are needed. Achieving complete knowledge of the various components of the ECM and understanding the multitudes of interactions and functions carried out by the intestinal ECM structure represents an important objective to unravel the mechanisms leading to the onset and the progression of several intestinal diseases related to alteration ECM remodeling, such as inflammatory bowel disease and its complications (intestinal fibrosis and cancer).

The relevance of complete knowledge of the ECM is underlying also by its increasing use in regenerative medicine, an innovative area based on the use of tissue engineering, stem cell biology, immunology, and bioengineering, to restore damaged tissues (231–240). Biomaterials used for this purpose may be distinct into synthetic and natural materials (231–233). The latter can be obtained or using the entire ECM or single components derived from ECM degradation such as collagen, laminin, glycosaminoglycans, and fibronectin (233). Among them, collagens and HA are the most widely used for biological folders (231). In the gastrointestinal tract, biological scaffolds represent a great promise as a new substrate to promote tissue remodeling *in situ* and replace damaged areas occurring for example during neoplasia and IBD and induce partial restoration and function of the organ. *In vivo* studies revealed that tubular porcine small intestine submucosa (SIS) bio-scaffold implantation can regenerate mucosa and smooth muscle in a damaged tissue after ileostomy (235). Few months after implantation, the neo-intestine presented restoration of the mucosa, smooth muscle, and serosa layers. Similarly, other authors reported that SIS bioscaffolds induced intestinal regeneration in an animal model of celiotomy (236). Following implantation, epithelium barrier renewal and regeneration of mucosa and submucosa layers were observed. ECM-derived scaffolds have proved to be effective also in the large intestine tract. Indeed, SIS biomaterials modulate the immune response in a rat model of colitis, reducing the local secretion of pro-inflammatory cytokines and enhancing the recovery of the colonic mucosa (237). Those mentioned are only a few examples of ECM bioscaffolds applications, but well-showed the importance of this innovative frontier, which could represent a promising therapeutic approach in many unresolved diseases (238, 239).

## Author Contributions

SP and GL performed the literature review, wrote the manuscript, and prepared the illustrations. RS and AV reviewed the manuscript and suggested important research points. EG reviewed the manuscript and provided critical comments. All authors have read and agreed to the published version of the manuscript.

## Conflict of Interest

The authors declare that the research was conducted in the absence of any commercial or financial relationships that could be construed as a potential conflict of interest.
